# Incidence and Risk Factors for Hypoxia in Deep Sedation of Propofol for Artificial Abortion Patients

**DOI:** 10.3389/fmed.2022.763275

**Published:** 2022-04-27

**Authors:** Yiling Fang, Yaru Xu, Silu Cao, Xiaoru Sun, Hui Zhang, Qi Jing, Li Tian, Cheng Li

**Affiliations:** ^1^Department of Anesthesiology and Perioperative Medicine, School of Medicine, Shanghai Fourth People’s Hospital, Tongji University, Shanghai, China; ^2^School of Medicine, Shanghai Fourth People’s Hospital, Translational Research Institute of Brain and Brain-Like Intelligence, Tongji University, Shanghai, China; ^3^Clinical Research Center for Anesthesiology and Perioperative Medicine, Tongji University, Shanghai, China; ^4^Department of Anesthesiology, Shanghai Tenth People’s Hospital, Tongji University School of Medicine, Shanghai, China; ^5^Department of General Practice, Zhangjiagang First People’s Hospital, Affiliated to Soochow University School of Medicine, Zhangjiagang, China

**Keywords:** deep sedation, propofol anesthesia, hypoxia, patient anxiety, painless artificial abortion

## Abstract

**Background:**

Respiratory depression is a life-threatening adverse effect of deep sedation. This study aimed to investigate the factors related to hypoxia caused by propofol during intravenous anesthesia.

**Methods:**

Three hundred and eight patients who underwent painless artificial abortion in the outpatient department of Shanghai Tenth People’s Hospital between November 1, 2019 and June 30, 2020 were divided into two groups according to whether the patients experienced hypoxia (SpO_2_ < 95%). Preoperative anxiety assessments, anesthesia process, and operation-related information of the two groups were analyzed. The univariate analysis results were further incorporated into logistic regression analysis for multivariate analysis to determine the independent risk factors affecting hypoxia.

**Results:**

Univariate analysis revealed that body mass index (BMI) (21.80 ± 2.94 vs. 21.01 ± 2.39; *P* = 0.038, 95% confidence interval (CI) = [−1.54, −0.04]), propofol dose (15.83 ± 3.21 vs. 14.39 ± 3.01; *P* = 0.002, CI = [−2.34, −0.53]), menopausal days (49.64 ± 6.03 vs. 52.14 ± 5.73; *P* = 0.004, CI = [0.79, 4.21]), State Anxiety Inventory score (51.19 ± 7.55 vs. 44.49 ± 8.96; *P* < 0.001, CI = [−9.26, −4.15]), and Self-rating Anxiety Scale score (45.86 ± 9.48 vs. 42.45 ± 9.88; *P* = 0.021, CI = [−6.30, −0.53]) were statistically significant risk factors for hypoxia during the operation. Logistic regression analysis showed that propofol dosage, menopausal days, and State Anxiety Inventory score were independent risk factors for hypoxia.

**Conclusion:**

Patient anxiety affects the incidence of hypoxia when undergoing deep intravenous anesthesia with propofol. We can further speculate that alleviating patient anxiety can reduce the incidence of hypoxia.

**Clinical Trial Registration:**

[http://www.chictr.org.cn], identifier [ChiCTR2000032167].

## Introduction

Abortion surgery is currently a means to terminate pregnancy within 14 weeks, and may cause severe discomfort to patients (1, 2). Globally, during the period 2015–2019, there were about 121 million unintended pregnancies each year, and about 73.3 million patients chose to miscarry (3). It is estimated that approximately 9 million people undergo abortion surgeries in China every year (4). Compared with traditional abortion surgeries, most painless abortions use propofol for deep sedation to reduce pain and increase comfort. Propofol is more effective than other anesthetics and has the pharmacokinetic characteristics of rapid action and smooth induction (5–10). However, in deep sedation, physical movement, respiratory depression, and drops in blood pressure are still widespread (11–14) of which respiratory depression is the most life-threatening (15). Painless abortions account for a large volume of operations and sedative adverse reactions occur in many cases, so it is necessary to conduct related research. There is no consensus about the probability of hypoxemia during general anesthesia for abortion in previous reports (16). Moreover, based on previous observations, we found that anxiety plays a decisive role in the occurrence of hypoxia in patients. In this study, we explored the incidence of hypoxia in abortion procedures under deep sedation and further explored its influencing factors, with a view to provide reference for clinical work.

## Materials and Methods

Ethical approval for this study (Number: SHSY-IEC-4.1/20-41/01) was provided by the Ethics Committee of Shanghai Tenth People’s Hospital, Shanghai, China on March 24th 2020 and successfully registered in the China Clinical Trial Registry (Number: ChiCTR2000032167).

A total of 308 patients who underwent painless artificial abortion in the outpatient department of Shanghai Tenth People’s Hospital from November 1st 2019 to June 30th 2020 were included in this study. A designated anesthesiologist collected the medical history and informed the patients and their families of the risks of surgery and anesthesia. After obtaining consent, the patients were screened according to the inclusion and exclusion criteria. The inclusion criteria included: (1) age between 18 and 45 years; (2) body mass index (BMI), 18–30 kg/m^2^; (3) 6–9 weeks of gestation; (4) American Society of Anesthesiologists (ASA) class I or II; (5) Mallampati class I or II; and (6) no coagulation dysfunction. The exclusion criteria included: (1) comorbidities such as motion sickness, hypertension, heart disease, asthma, epilepsy, Parkinson’s disease, depression, etc.; (2) previous history of drug allergies; and (3) patients with conventional blood oxygen saturation less than 98%.

In each operating room, a gynecologist, anesthesiologist, nurse, and recorder were present. All patients fasted for 8 h and did not drink fluids for 4 h before the operation. The medical history and inspection report of the patients were collected 1 h before the operation (including age, height, weight, amenorrhea days, whether smoking and drinking, etc.).

All patients were assessed for anxiety 30–60 min before surgery. The State-Trait Anxiety Inventory (STAI) and Self-rating Anxiety Scale (SAS) tools were used to determine patient anxiety. In these validated tools, higher scores indicate higher degrees of anxiety (17–19). The state-STAI (S-STAI) assesses the current state of anxiety. The specific time is directly related to the psychological and physiological response. The trait-STAI (T-STAI) refers to personality traits. Assessing the individual differences of “anxiety” is relatively stable, description and presents the current state anxiety tendency related to individual differences (20).

Anesthesiologists were given information regarding routine monitoring, venipuncture and catheterization, measurement of blood pressure (preoperative, intraoperative 1 min, 5 min), heart rate, oxygen saturation, and the baseline bispectral index (BIS) score. The patient’s mask administered oxygen 6 L/min (37°C, oxygen concentration 100%). The patient then received an intravenous bolus of butorphanol tartrate 0.01 mg/kg for pain and propofol 2 mg/kg for induction of anesthesia, and the operation started after the ciliary reflex disappeared. In case of intraoperative body movement, narcotic analgesic propofol 0.5 mg/kg was administered. The aim was to maintain the BIS value between 50 and 70. Sedation-related adverse events were defined as systolic blood pressure lower than 80 mmHg, heart rate lower than 50 beats/min, hypoxia (75% ≤ SpO_2_ < 90%, duration <60 s), and severe hypoxia (SpO_2_ < 75%, or 75% ≤ SpO_2_ < 90%, lasting ≥60 s). In case of adverse effects, norepinephrine 20–40 μg was administered to patients with hypotension and intravenous atropine 0.25–0.5 mg to patients with bradycardia. When SpO_2_ became <95%, we determined that the patient was likely hypoxic. The jaw was first lifted to open the airway and increase oxygen flow. Then, ambu-bag ventilation was used to correct the hypoxia. If hypoxia could not be corrected by ambu-bag ventilation, tracheal intubation was performed. All sedation in this study was administered by an anesthesiologist.

The lowest blood oxygen saturation and hypoxia incidence were recorded. Between the hypoxic and non-hypoxic groups, we compared age, weight, gestational age, the number of childbirths, the amount of medication, history of abortion, STAI score, SAS score, and other factors. We also recorded the patient’s operation time, which was defined as the time from the disappearance of the ciliary reflex to the time the patient opened her eyes.

According to the preliminary experiment, considering that the overall occurrence of hypoxia (SpO_2_ < 95%) is about 20%, we included 308 patients with consideration for a shedding rate of 20%. The SPSS 20.0 software (IBM SPSS Inc., Chicago, United States) was used for processing and analysis. The basic data were first tested for normal distribution. Data that were normally distributed were represented by the mean ± SD, and the data that were not normally distributed were represented by median (P25, P75). The two-sample *t*-test was used for continuous variables, Chi-square test for categorical variables, and logistic regression analysis for multivariate analysis. The difference was statistically significant when *P* < 0.05.

## Results

**FIGURE 1:**
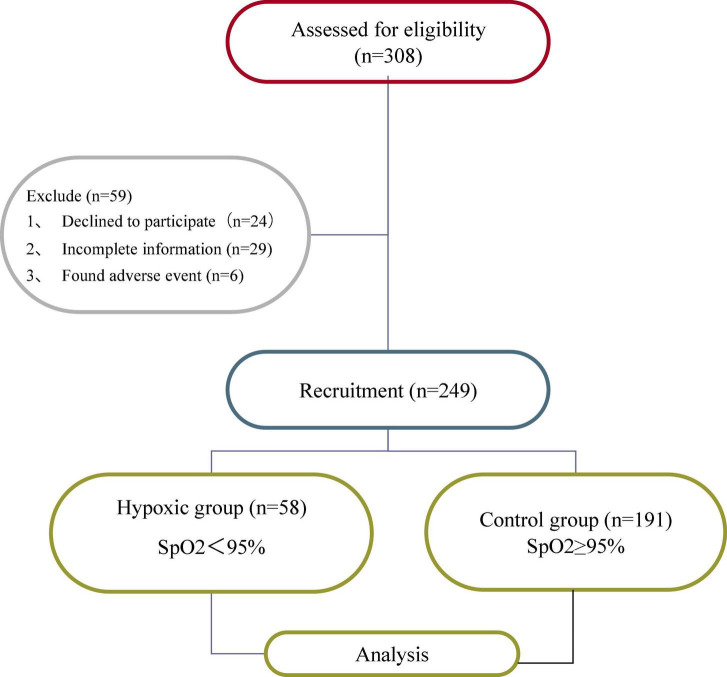
Flow diagram showing enrolled and excluded patients.

Fifty-nine of the 308 patients were excluded due to refusal to participate (*n* = 24), incomplete information (*n* = 29), or adverse events (*n* = 6), leaving 249 patients for analysis (Figure 1).

Of the 249 participants who completed the study, 58 patients (23.3%) developed hypoxia. Comparison between the hypoxia and non-hypoxia groups showed that there was no significant difference in age, height, operation time, physical activity, smoking and drinking history, and T-STAI scores (*P* > 0.05). The BMI (21.80 ± 2.94 vs. 21.01 ± 2.39; *P* = 0.038, 95% confidence interval (CI) = [−1.54, −0.04]), propofol dose (15.83 ± 3.21 vs. 14.39 ± 3.01; *P* = 0.002, CI = [−2.34, −0.53]), and menopausal days (49.64 ± 6.03 vs. 52.14 ± 5.73; *P* = 0.004, CI = [0.79, 4.21]), S-STAI score (51.19 ± 7.55 vs. 44.49 ± 8.96; *P* < 0.001, CI = [−9.26, −4.15]), and SAS score (45.86 ± 9.48 vs. 42.45 ± 9.88; *P* = 0.021, CI = [−6.30, −0.53]) were found to be risk factors for patients with hypoxia (Table 1).

**TABLE 1:** Single factor analysis of influencing factors of hypoxia in patients undergoing painless abortion.

Factors	Hypoxic group (*n* = 58)	Control group (*n* = 191)	*P*-value
Age (years)	31.79 ± 5.89	30.80 ± 6.35	0.290
Height (cm)	161.48 ± 4.75	161.84 ± 4.43	0.600
BMI (kg/m^2^)	21.80 ± 2.94	21.01 ± 2.39	0.038
Propofol dosage (ml)	15.83 ± 3.21	14.39 ± 3.01	0.002
Operation time (s)	468.02 ± 184.86	454.08 ± 141.15	0.542
Menopausal days (days)	49.64 ± 6.03	52.14 ± 5.73	0.004
**Body movement [*n* (%)]**			
Yes	25 (43.1)	93 (48.7)	
No	33 (56.9)	98 (51.3)	0.455
**Smoking [*n* (%)]**			
Yes	5 (8.6)	18 (9.4)	
No	53 (91.4)	173 (90.6)	0.853
**Drinking [*n* (%)]**			
Yes	4 (6.9)	10 (5.2)	
No	54 (93.1)	181 (94.8)	0.744
S-STAI score (points)	51.19 ± 7.55	44.49 ± 8.96	<0.001
T-STAI score (points)	43.21 ± 8.76	42.07 ± 8.70	0.386
SAS score (points)	45.86 ± 9.48	42.45 ± 9.88	0.021

*BMI, body mass index; S-STAI, State Anxiety Inventory; S-STAI, Trait Anxiety Inventory; SAS, Self-Rating Anxiety Scale.*

Multivariate analysis was used in order to exclude the influence of confounding factors. The results showed that BMI and SAS scores were not independent risk factors for hypoxia, while propofol dosage, menopausal days, and S-STAI score were independent risk factors (Table 2).

**TABLE 2:** Logistic regression analysis.

		95% CI		
Variable	OR	Upper limit	Lower limit	*P*-value
Propofol dosage	1.267	1.113	1.443	<0.001
BMI	1.137	0.987	1.31	0.076
Body movement	2.445	1.138	5.255	0.022
Menopausal days	0.929	0.875	0.986	0.015
T-STAI score	1.181	1.111	1.256	<0.001
S-STAI score	0.906	0.855	0.961	0.001
SAS score	1.038	0.993	1.085	0.096

*OR, odds ratio; CI, confidence interval; BMI, body mass index; S-STAI, State Anxiety Inventory; T-STAI, Trait Anxiety Inventory; SAS, Self-Rating Anxiety Scale.*

**FIGURE 2:**
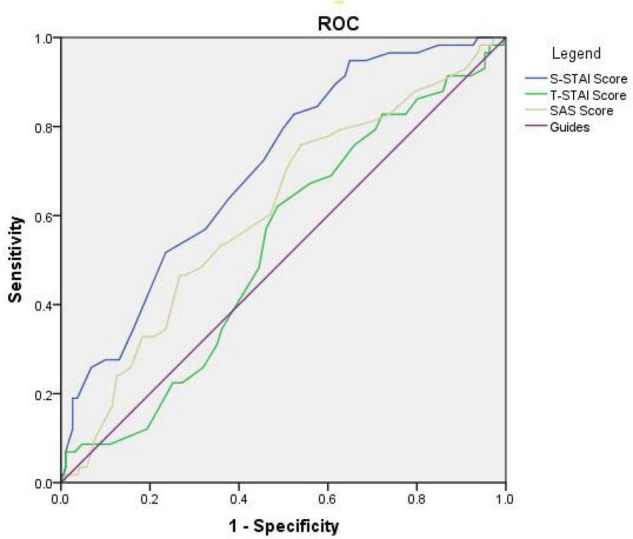
Receiver operating characteristic (ROC) curve of the final logistic regression model of three anxiety scores associated with hypoxia. The curve is expressed as a solid line. AUC, area under the curve; ROC, receiver operating characteristic.

According to Figure 2, the AUC value corresponding to the S-STAI score was 0.706, indicating that the S-STAI score has a higher diagnostic value for patients with hypoxia. The AUC value corresponding to the T-STAI score is 0.528 (*P* = 0.386) indicating that it has no diagnostic value for patients with hypoxia. The AUC value corresponding to the SAS score is 0.609 (*P* = 0.021) indicating low diagnostic value.

## Discussion

In this study, we investigated the incidence of hypoxia after propofol sedation during painless abortion procedures in 249 patients; of these, 58 patients developed hypoxia. Univariate analysis revealed that BMI, propofol dose, days of amenorrhea, S-STAI score, and SAS score were significant risk factors for hypoxia. Multivariate logistic regression analysis showed that propofol dosage, menopausal days, and S-STAI score were independent risk factors for hypoxia. As days of amenorrhea cannot be changed in patients, early intervention is crucial for reducing hypoxia in patients who receive large doses of propofol and have high S-STAI scores. We recommend using the STAI and SAS tools to determine patient anxiety and providing early intervention and focused attention to those with high scores to reduce the occurrence of hypoxia during deep sedation. A simple preoperative anxiety scale assessment alone does not add significantly to the clinician’s workload, but the benefits to the patient can be substantial.

There is some consensus in literature on the risk factors for adverse events related to deep sedation with propofol. Geng et al. reported that age, BMI, total propofol dose, smoking, and alcohol consumption are adverse risk factors associated with gastrointestinal endoscopy hypoxemia (21). Another study suggested that age, larger propofol doses, and smoking are associated with a higher incidence of sedation-related adverse events during colonoscopy (22). Our data show that propofol dosage and anxiety are related to hypoxia during painless abortion surgery. However, our study population only consisted of women, with a relatively small proportion of those who smoked. In addition, the aforementioned previous studies did not perform patient anxiety assessments and did not consider the influencing factors of anxiety. Therefore, our inclusion of anxiety scores is innovative.

Research shows that most people have preoperative anxiety due to anticipation of pain and risks, especially female patients (23). It is estimated that about 60–80% of patients undergoing implantable cardioverter defibrillator (ICD) placement have anxiety before surgery (24); among patients undergoing coronary surgery, 44.5% of patients have anxiety symptoms. Additionally, the anxiety in female patients is significantly higher than that in male patients (25). Evidently, female patients are more likely to suffer from anxiety prior to surgeries, such as abortion. In the past, some people were believed to be naturally more prone to symptom of fear and discomfort (26), and they had anxiety when encountering difficulties. Mild preoperative anxiety can adjust physical functions and help patients through the perioperative period. However, patients with high levels of anxiety before surgery are prone to hyperventilation, and PetCO_2_ is low, which is more likely to cause a vasovagal incident (27). In addition, hyperventilation may cause apnea or even reflex cardiac arrest (28, 29). Preoperative anxiety is an important predictor of propofol dosage (30, 31). Patients with higher anxiety scores require a larger dose of propofol to achieve mild sedation (32). However, the increased dose of propofol is also a major risk factor leading to hypoxia. Studies have shown that propofol can cause respiratory depression by inhibiting the neuromodulation of the respiratory system, especially the maintenance of upper airway patency and the reflex related to the control of the patency the chemically sensitive upper airway (33). In general, the larger the dose, the stronger the inhibitory effect. In surgery where the propofol dose is relatively small, the incidence of adverse reactions and complications is low (34).

In our study, we found that the smaller the gestational week, the greater the likelihood of hypoxia. Previous studies have shown that from early pregnancy up to the second trimester, the cardiac output of pregnant women continues to increase and remains stable thereafter (35), which can increase the blood oxygen concentration and reduce the occurrence of hypoxia.

Regardless of the anesthetic agent used, adverse effects of hypoxia are more likely to occur during sedation (36–38). Prolonged hypoxia can cause damage to target organs, including acute kidney injury, pulmonary edema, myocardial infarction, acute respiratory distress syndrome, and ultimately death or residual disability (39). The mortality rate of severely ill patients with hypoxemia is 27%, which is 15% higher than that of severely ill patients without hypoxemia (40). Although the application of propofol to general anesthesia resulted in deaths due to severe hypoxia, there were not many cases of death caused by severe hypoxia alone. However, in a retrospective cohort study of 2937 operations performed by Clemens, severe hypoxia did not respond to treatment and resulted in termination of the operation (12, 41). Klare et al. examined the incidence of hypoxemia during endoscopic retrograde cholangiopancreatography sedation with midazolam and propofol and observed one death event due to severe hypoxemia (42). Although the incidence is not high, the number of people receiving general anesthesia for abortions is huge. Therefore, it is particularly important to reduce the occurrence of hypoxia during general anesthesia, especially during painless abortion surgery.

This study had some limitations. First, we recruited a small sample of patients; so, the number of patients who experienced hypoxia was also less. Second, there might have been differences in the techniques of different gynecological surgeons, even if they underwent uniform training, resulting in differences in proficiency. In clinical trials, it was found that obese patients were more prone to hypoxia (43, 44). In our univariate analysis, BMI was an influencing factor, but the multivariate analysis showed no statistical significance, which may be caused by the low sample size. Although it is established that preoperative anxiety is an important factor influencing the occurrence of hypoxia during deep sedation, practical ways to reduce it have still not been found. The main causes of preoperative anxiety are as follows: fear of the surgical unknown, fear of the disease, and fear of the end of life (45). Severe anxiety often causes intraoperative hemodynamic problems and hampers the course of recovery (46). Previous studies have shown that people under 30 years of age and women are more likely to have higher levels of anxiety (47). A study suggests that musical interventions may provide a viable alternative to sedatives and anti-anxiety medications to reduce preoperative anxiety (48). Previous studies have shown that relaxation therapy is well able to counter the effects of stress and thus reduce sympathetic activity. Music therapy and progressive muscle training both have good synergistic effects in reducing preoperative anxiety (44). Relaxation therapy is an effective non-pharmacological treatment for anxiety relief, easy to implement, and has high compliance. We will evaluate in a follow-up study whether relaxation therapy can reduce the occurrence of hypoxia in anxious patients undergoing painless abortion surgeries.

## Conclusion

Hypoxia is one of the most common side effects of a painless abortion procedure under propofol anesthesia. Patient anxiety is important factor affecting deep sedation with propofol.

## Data Availability Statement

The original contributions presented in the study are included in the article/supplementary material, further inquiries can be directed to the corresponding author.

## Ethics Statement

The studies involving human participants were reviewed and approved by the Ethics Committee of Shanghai Tenth People’s Hospital, Shanghai, China. The patients/participants provided their written informed consent to participate in this study.

## Author Contributions

YF: methodology, validation, visualization, writing – original draft, and writing – review and editing. YX: investigation, formal analysis, and writing – review and editing. SC: investigation and visualization. XS: validation and writing – review and editing. HZ: software and formal analysis. QJ: resources and investigation. LT: conceptualization, supervision, and writing – review and editing. CL: conceptualization, methodology, resources, investigation, formal analysis, writing – original draft, writing – review and editing, project administration, and funding acquisition. YF and YX contributed equally to this work. All authors contributed to the article and approved the submitted version.

## Conflict of Interest

The authors declare that the research was conducted in the absence of any commercial or financial relationships that could be construed as a potential conflict of interest.

## Publisher’s Note

All claims expressed in this article are solely those of the authors and do not necessarily represent those of their affiliated organizations, or those of the publisher, the editors and the reviewers. Any product that may be evaluated in this article, or claim that may be made by its manufacturer, is not guaranteed or endorsed by the publisher.
